# Translation, cross-cultural adaptation, and validation of the 10-item spine functional index (SFI-10) in the Brazilians with musculoskeletal spine disorders

**DOI:** 10.1186/s12891-024-07406-0

**Published:** 2024-04-05

**Authors:** Devyd Weyder do Nascimento Freitas, Almir Vieira Dibai-Filho, André Pontes-Silva, Gabriel Gardhel Costa Araujo, Augusto Ribeiro de Oliveira, Plinio da Cunha Leal, Charles Philip Gabel, Cid André Fidelis-de-Paula-Gomes, Christian Emmanuel Torres Cabido

**Affiliations:** 1 Postgraduate Program in Physical Education, Universidade Federal do Maranhão, São Luís, MA Brazil; 2 Postgraduate Program in Physical Therapy, Universidade Federal de São Carlos, São Carlos, SP Brazil; 3 Access Physiotherapy, Coolum Beach, QLD Australia; 4 Postgraduate Program in Rehabilitation Sciences, Universidade Nove de Julho, São Paulo, SP Brazil

**Keywords:** Spine, Chronic pain, Patient health questionnaire, Measurement properties

## Abstract

**Purpose:**

To translate and cross-culturally adapt the Spine Functional Index (SFI) into Brazilian Portuguese (SFI-Br) in individuals with musculoskeletal spine disorders.

**Methods:**

Participants (*n*=194) answered the Numerical Pain Rating Scale (NPRS), 36-item Short-Form Health Survey (SF-36), Roland-Morris Disability Questionnaire for General Pain (RMDQ-g), and SFI-25 incorporating the SFI-10. Structural validity, from confirmatory factor analysis (CFA), used comparative fit index (CFI), Tucker-Lewis index (TLI), root mean square error of approximation (RMSEA), and chi-square/degrees of freedom (DF). The best structure was considered from the lower values of the Akaike Information Criterion (AIC) and Bayesian Information Criterion (BIC). Construct and criterion validity used Spearman’s correlation coefficient (rho). Internal consistency used Cronbach’s alpha, reliability used intraclass correlation coefficient (ICC_2,1_), with ceiling and floor effects determined. Error used the standard error of the measurement (SEM) and minimal detectable change, 90% level (MDC_90_).

**Results:**

Adequate fit indices demonstrated an unequivocal one-factor structure only for the SFI-10 (chi-square/DF <3.00, CFI and TLI >0.90, RMSEA <0.08). The SFI-10-Br correlation was high with the SFI-Br (rho=0.914, p<0.001), moderate for the RMDQ-g (rho=-0.78), SF-36 functional capacity domain (rho=0.718) and NPRS (rho=-0.526); and adequate for the remaining SF-36 domains (rho>0.30). Test-retest reliability (ICC_2,1_=0.826) and internal consistency (alpha=0.864) were high. No ceiling or floor effects were observed, and error was satisfactory (SEM=9.08%, MDC_90_=25.15%).

**Conclusion:**

The SFI Brazilian version was successfully produced with the 10-item version showing an unequivocal one-factor structure, high construct and criterion validity, reliability, internal consistency, and satisfactory error. Further research on responsiveness is required.

**Supplementary Information:**

The online version contains supplementary material available at 10.1186/s12891-024-07406-0.

## Introduction

Currently, at least 43 patient-reported outcome measures (PROMs) have been developed to assess problematic aspects related to the spine [1]. Of these, the most commonly used for lumbar problems are the Roland Morris Disability Questionnaire (RMDQ) [2] and Oswestry Disability Index (ODI) [3], and for cervical problems the Neck Disability Index (NDI) [3]. These PROMs represent the vast majority of all spine research results, have the most cross-cultural adaptations, and are consequently the most widely reported PROMs in the spine-specific literature. However, despite strong advocacy, they do not assess spine functionality as a single kinetic chain and are not applicable to the same patient that suffers symptoms in different spine regions [4].

To date, six whole-spine PROMs have been proposed to assess the different spine regions: the Functional Rating Index (FRI) [5], the Bournemouth Questionnaire [6], the Extended Aberdeen Spine Pain Scales [7], the Pain Disability Questionnaire [8], the Core Outcome Measures Index [9], and the Spine Functional Index (SFI). However, none of these have demonstrated an unequivocal one-dimensional factor structure through robust analyses such as Rasch and/or factor analysis [1]. The SFI has verified one-dimensionality using exploratory factor analysis (EFA) which was inconclusive on confirmatory factor analysis (CFA) due to the analysis methodology employed [10, 11].

Consequently, there remains a need for non-condition-specific whole-spine PROMs with adequate measurement properties, particularly factor structure. The SFI, as a non-specific instrument, was designed to overcome the limitations of PROMs that are only suited to a single condition or spine region. The SFI has adequate measurement properties and has been used in various populations and age groups. It contains 25 items with a three-point Likert scale response option (per item) and has been translated and validated into Spanish [12], Persian [10], Korean [13], Turkish [14], Chinese [11], and Polish [15]. It was included in a whole-spine systematic review [4] and has demonstrated favorable responsiveness and error determination in a chronic neck pain population [16]. Nevertheless, the SFI still lacks translation and cross-cultural adaptation for Brazilian populations and independent clarification of the factor structure with appropriate statistical analysis.

Therefore, the purpose of this study was to translate, cross-culturally adapt, and validate the SFI into Brazilian Portuguese (SFI-Br) in individuals with musculoskeletal spine disorders. In addition, the determination of the psychometric properties of structural validity for the 25-item and the shortened 10-item (SFI-10-Br) versions, then if valid to continue with construct and criterion validity, plus test-retest reliability, internal consistency, and error.

## Methods

### Setting and ethical aspects

A cross-sectional questionnaire validation study was developed according to the guidelines for the process of cross-cultural adaptation of self-report measures [17] and the consensus-based standards for the selection of health measurement instruments (COSMIN) [18, 19]. Permission to conduct the validation of the SFI in Brazilian Portuguese was granted by the questionnaire’s authors.

The study was conducted in the city of São Luís (Brazil) and was designed in two phases: I) translation and adaptation of the SFI into Brazilian Portuguese, II) then subsequent validation of the final version in both the 25-item and shortened (SFI -10) version. All procedures were approved by the Research Ethics Committee of the Universidade Federal do Maranhão (report number 4.284.203).

### Study size and sampling

In factorial analysis, the guidelines recommend the sample size be seven times the number of questionnaire items [20]. Since the SFI has 25 items, the minimum sample size was 175 participants, however to test the pre-final version of the SFI, 30 participants were sampled [17]. To test validity, the final cross-culturally adapted SFI-version was administered to 194 participants. For reliability analysis, a subsample was assessed twice within seven days during a period of no treatment [20]. The subsample was to include only participants who reported a pain level >3 after seven days [21, 22], as such, it consisted of 43 participants.

### Participants eligibility criteria

Participants were recruited who had chronic pain and musculoskeletal dysfunction in the spine of duration ≥3 months and pain intensity ≥3 on the Numerical Pain Rating Scale (NPRS) [23]. Eligible participants were required to be competent in reading and writing Brazilian Portuguese, had no medical diagnosis or cognitive dysfunction, and were ≥18 years of age. Participants with any history of surgery <6 months ago, the presence of inflammatory or infectious disease, neurological disorders, cancer, and severe psychiatric disorders were excluded [24].

### Assessments and tools

The survey was conducted online during the COVID-19 pandemic (2020-2021) using the Google Forms platform (Mountain View, CA, USA). Initial recruitment used social networks and messaging apps (WhatsApp and Instagram, Meta, Menlo Park, CA, USA). Volunteers contacted a physiotherapist who sent a link with all survey information and participants completed the survey independently.

The data was extracted in a controlled manner to eliminate duplicate responses (e-mail, name, age, and phone number verification). All study participants provided informed consent in an electronic format and completed the NPRS, the 36-item Short-Form Health Survey (SF-36), the Roland-Morris Disability Questionnaire for General Pain (RMDQ-g), the SFI, and questions on clinical and demographic characteristics.

### Questionnaires

The NPRS is a simple 11-point measurement (0 to 10), where 0 represents “no pain” and 10 represents “the worst pain imaginable”. Individuals rated their pain based on these parameters [23]. Although some culturally adapted SFI versions did not concurrently use the NPRS, it was included in this study as a self-reported screening measure to quantify pain intensity and enroll participants.

The SF-36 assesses eight health domains: functional capacity, physical limitation, pain, general health status, vitality, social aspects, emotional aspects, and mental health. Scale scores are calculated by summing the responses of the scale items then converting the raw score of each domain into a ‘Health status’ percentage value (0-100%) where 0 represents the ‘Worst’ and 100 represents the ‘Best’. The SF-36 has already been culturally adapted and validated for Brazilian Portuguese [25].

The RMDQ-g was already validated and adapted for the Brazilian population with generalized pain [25]. It is a 24-item instrument with a binary response option where each item describes daily activities related to physical function to specifically assess disability associated with chronic pain in general. Each selected item is quantified with a score of 1, so that the total score ranges from 0 to 24. The higher the total score, the greater the disability.

The SFI is a 25-item instrument that describes symptoms and difficulties commonly experienced by people with spine disorders. The questionnaire concurrently assesses the function of the neck, thoracic and lumbar regions, allowing it to be used in a variety of musculoskeletal conditions. It has a three-point Likert scale response option for each item, as follows: “Yes” equals a score of 1, “Partially” equals a score of 0.5, and “No” equals a score of 0. It takes approximately 2.5 minutes to complete and score. The 25 items are summated to give the raw score, which is multiplied by four and then subtracted from 100 to produce a percentage value. The higher the score, the better the spine function [1]. In contrast to other SFI validation studies, this study also tested a shortened version with 10-items (items 3, 6, 10, 11, 12, 13, 17, 20, 22, and 24), where higher values indicate better column functionality (i.e., 100 – [Total × 10]) [26]. The SFI-10-Br is available at http://questionariosbrasil.blogspot.com/.

### Translation and adaptation

Forward translation to Brazilian Portuguese was completed by two independent translators: a physiotherapist with 10 years of experience; and an English teacher with 22 years of experience in translation without technical knowledge of health-related subjects. Both translators were native Brazilian Portuguese speakers and fluent in English. Following subsequent discussion and revisions the two translators produced a synthesized consensus version under the supervision of the lead researcher which was approved by those involved in the research. Back translation was completed by two independent native English-speaking translators with Portuguese fluency and no technical health knowledge. To arrive at a pre-final consensus SFI-Br, an expert committee was formed that included four rehabilitation specialists and the four translators.

To test the pre-final SFI-Br 30 first language Brazilian Portuguese speaking individuals with spine disorders were recruited. The participants read and completed the questionnaire then provided feedback on their understanding of each item-question with “Yes” and “No” responses. All questions not understood by >20% of participants were reworded and re-tested in a new sample (*n*=30). This process was repeated until the required response level for understanding was reached, thus establishing the final SFI-Br version and ensuring face and content validity [27].

### Statistical methods

Structural validity was performed through confirmatory factor analysis (CFA) with R Studio software (Boston, MA, USA), using the Lavaan and semPlot packages. The analysis was based on a polychoric covariance matrix and robust diagonally weighted least squares (RDWLS) extraction due to the SFI being ordinal categorical in nature. The following indices checked the model fit: comparative fit index (CFI), Tucker-Lewis index (TLI), root mean square error of approximation (RMSEA), and chi-square/degrees of freedom (DF). For model acceptance parameters the CFI and TLI >0.90, RMSEA and SRMR <0.08, and Chi-square/DF <3 were considered [28]. To compare the SFI factor structures, adequacy from the lower value of the Akaike Information Criterion (AIC) and the Bayesian Information Criterion (BIC) was considered.

For construct and criterion validity, after applying the Kolmogorov-Smirnov normality test and identifying a non-normal distribution, the Spearman’s correlation coefficient (rho) was used to test the magnitude of the correlation between the SFI-Br and the concurrently completed criteria. The hypothesis of this analysis was that: the SFI correlation magnitude with the RMDQ-g and the SF-36 functional capacity domain (similar constructs) would be >0.50; and correlation with the NPRS and SF-36 would be rho>0.30 to 0.50 (related constructs) [18]. Correlation between the SFI-Br and the SFI-10-Br (criterion validity) would be considered adequate at rho >0.70 [18].

To evaluate reliability, participants completed the SFI twice: once as a test and again after a 7-day interval as a retest. As such, reliability was determined from a test-retest model calculating the intraclass correlation coefficient (ICC_2,1_) with >0.75 considered adequate [29]. Internal consistency used Cronbach’s alpha to determine whether there were heterogeneous or redundant items in the questionnaire with adequacy at a cut off range of >0.70 to <0.95 [28]. The standard error of the measurement (SEM) and the minimum detectable change at the 90% level (MDC_90_) are directly determined by the reliability and were subsequently calculated [30].

For ceiling and floor effects, which are present when >15% of study participants reach the minimum or maximum values of total questionnaire score, a problem in the capacity to assess the instruments responsiveness is indicated. The descriptive analysis was performed using SPSS software, version 17.0 (Chicago, IL, USA), and described the variables in terms of mean and standard deviation (SD) or absolute and relative frequencies.

## Results

### Translation, cross-cultural adaptation, and sample characterization

The pre-final SFI-Br (*n*=30) was administered to individuals with spinal musculoskeletal disorders and demonstrated that all item-questions were understood by >80% of participants. A total of *n*=214 participants were recruited to the study with *n*=20 excluded as pain intensity was <3/10. The final sample was *n*=194 subjects with a sub-sample of *n*=43 for reliability.

The sample mean age was 29.11 years (SD = 8.44) with a mean chronic pain duration of 48.70 months (SD = 57.3). The majority of participants were female, single, and physically active. Other demographic and clinical characteristics are described in Table 1. The structures of the SFI-Br and SFI-10-Br tested in this study are described in Table 2 with the scores of the questionnaires used in this study described in Table 3.

**Table 1:** Demographic and clinical characteristics of the total sample (*n* = 194)

**Variables**	**Absolute number (%)**
Sex (female)	136 (70%)
Marital status	
Single	59 (30%)
Married	128 (66%)
Divorced	7 (4%)
Level of education	
Complete primary education	2 (1%)
Incomplete secondary education	1 (1%)
Complete secondary education	19 (10%)
Incomplete higher education	54 (28%)
Complete higher education	53 (27%)
Complete post-graduate	41 (21%)
Incomplete post-graduate	24 (12%)
Physical Activity (yes)	110 (57%)
Affected region	
Neck	38 (20%)
Thoracic	60 (31%)
Low back	96 (49%)

**Table 2:** Versions of the Spine Functional Index Questionnaire tested

**Items**	**25 items**	**10 items**
1. I stay at home most of the time	Yes	Not
2. I change position frequently for comfort.	Yes	Not
3. I avoid heavy jobs (e.g., cleaning, lifting more than 5kg or 10lbs, gardening, etc.).	Yes	*Yes*
4. I rest more often.	Yes	Not
5. I get others to do things for me.	Yes	Not
6. I have the pain/problem almost all the time.	Yes	*Yes*
7. I have difficulty lifting and carrying (e.g., bags, shopping up to 5kg or 10lbs).	Yes	Not
8. My appetite is now different.	Yes	Not
9. My walking or normal recreation or sporting activity is affected.	Yes	Not
10. I have difficulty with normal home or family duties and chores.	Yes	*Yes*
11. I sleep less well.	Yes	*Yes*
12. I need assistance with personal care (e.g., washing and hygiene).	Yes	*Yes*
13. My regular daily activities (work, social contacts) are affected.	Yes	*Yes*
14. I am more irritable and/or bad tempered.	Yes	Not
15. I feel weaker and/or stiffer.	Yes	Not
16. My transport independence is affected (driving, public transport).	Yes	Not
17. I require assistance or am slower with dressing.	Yes	*Yes*
18. I have difficulty moving in bed.	Yes	Not
19. I have difficulty concentrating and/or reading.	Yes	Not
20. My sitting is affected.	Yes	*Yes*
21. I have difficulty getting in and out of chairs.	Yes	Not
22. I only stand for short periods of time.	Yes	*Yes*
23. I have difficulty squatting and/or kneeling down.	Yes	Not
24. I have trouble reaching down (e.g., pick-up things, put on socks).	Yes	*Yes*
25. I go up stairs slower or use a rail.	Yes	Not

**Table 3:** Questionnaire scores (*n*=194)

**Questionnaires**	**Mean**	**SD**	**Median**	**Minimum**	**Maximum**
SFI-25	70.36	18.19	74	6	98
SFI-10	73.15	19.46	75	10	100
NPRS	5.86	1.84	6	3	10
RMDQ-g	5.78	5.46	4	0	24
SF-36					
Functional capacity	70.07	22.81	70	10	100
Physical limitation	58.76	40.72	75	0	100
Pain	35.78	9.25	32	10	64
General health status	49.73	17.74	52	10	100
Vitality	41.90	21.05	40	0	95
Social aspects	65.56	72.53	62,50	0	1000
Emotional aspects	55.15	42.78	66,70	0	100
Mental health	53.85	42.78	56	0	100

*SFI* Spine Functional Index, *NPRS* Numerical Pain Rating Scale, *RMDQ-g* Roland-Morris Disability Questionnaire for general pain, *SF-36* 36-Item Short-Form Health Survey questionnaire

SFI Brazilian version was successfully produced with the 10-item version showing an unequivocal one-factor structure, high construct and criterion validity, reliability, internal consistency, and satisfactory error.

### Structural, construct, and criterion validity

**Fig. 1:**
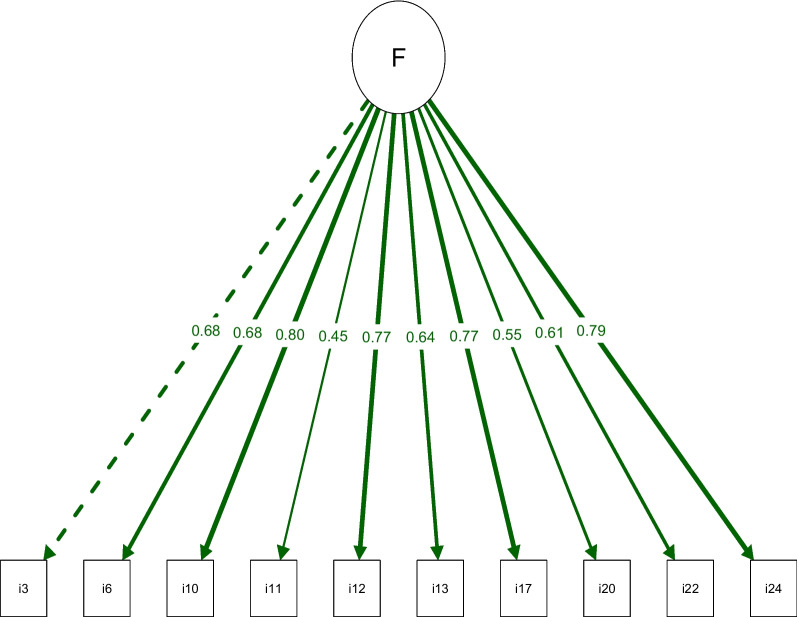
Path diagram of the 10-item Brazilian version of the SFI with values representing the factor loadings between the domain and each item.

Two internal structure options were tested for the SFI-Br: model 1, with one domain and 25 items, based on the original SFI; and model 2, with one domain and 10 items (items 3, 6, 10, 11, 12, 13, 17, 20, 22, and 24), as determined for the shorted version SFI-10. The comparison between the two models (SFI-Br versus SFI-10-Br) is detailed in Table 4. Adequate fit indices were only observed in the SFI-10-Br version (Chi-square/DF <3.00, CFI and TLI >0.90, RMSEA <0.08). Further, the short version had the lowest AIC and BIC values with corresponding factor loadings (>0.40) (see Fig. 1). Consequently, as only the SFI-10-Br demonstrated one-dimensionality, further results are only reported for the shortened version.

**Table 4:** Comparison between the long and short structures of the Spine Functional Index (SFI)

**Structures**	**Chi-square/DF**	**CFI**	**TLI**	**RMSEA (90% CI)**	**AIC**	**BIC**
25 items	1.94	0.896	0.887	0.070 (0.061, 0.079)	1867.763	2031.156
10 items	1.88	0.959	0.947	0.068 (0.042, 0.093)	635.275	700.632

*CFI* Comparative Fit Index, *TLI* Tucker-Lewis index, *RMSEA* Root Mean Square Error of Approximation, *CI* confidence interval, *AIC* Akaike Information Criterion, *BIC* Bayesian information criterion

The construct validity, from the SFI-10-Br correlation coefficient with the RMDQ-g (rho=-0.777) and the SF-36 functional capacity domain (rho=0.718) (similar constructs), was adequate as it exceeded the cut off (rho>0.50), as did the NPRS (rho=-0.526). Correlations with the remaining SF-36 domains was adequate according to the a-priori hypothesis (rho>0.30). Criterion validity between the SFI-Br and SFI-10-Br versions was strong (rho=0.914, p<0.001) (Table 5).

**Table 5:** Correlation among Spine Functional Index (SFI) with 10 items and other instruments.

**Instruments**	**SFI-10**
Numerical Pain Rating Scale	rho = -0.526, *p* <0.001
RMDQ-g	rho = -0.777, *p* <0.001
SF-36	
Functional capacity	rho = 0.718, *p* <0.001
Physical limitation	rho = 0.577, *p* <0.001
Pain	rho = 0.630, *p* <0.001
General health status	rho = 0.493, *p* <0.001
Vitality	rho = 0.439, *p* <0.001
Social aspects	rho = 0.434, *p* <0.001
Emotional aspects	rho = 0.485, *p* <0.001
Mental health	rho = 0.459, *p* <0.001

*SFI* Spine Functional Index, *RMDQ-g* Roland-Morris Disability Questionnaire for general pain, *SF-36* 36-Item Short-Form Health Survey questionnaire

### Reliability, ceiling and floor effects, and error

There was high test-retest reliability (ICC_2,1_=0.826) and internal consistency (alpha=0.864). No ceiling or floor effects were observed (minimum, *n*=0 or 0%; maximum, *n*=15 or 7.7%). The SEM and MDC values are described in the Table 6.

**Table 6:** Reliability and internal consistency of the Spine Functional Index (SFI) with 10 items

**Variable**	**Value**
Mean (standard deviation)	
Test	75.54 (17.03)
Retest	78.83 (15.99)
Intraclass correlation coefficient	0.826
Confidence interval at 95%	0.701, 0.902
Standard error of measurement	
Score	7.00
%	9.08
Minimum detectable change	
Score	19.42
%	25.15
Cronbach’s alpha	0.864

## Discussion

### Main results synthesis

The questionnaire was successfully translated and culturally adapted as it proved to be easy to interpret with no reported misunderstanding of items by >20% of participants [27]. In addition, the shortened SFI 10-item version showed the preferred structural validity through an unequivocal one-dimensional factor structure. There was adequate correlation with the RMDQ-g and SF-36 functional capacity domain (similar constructs), and the remaining criteria (related constructs). Additionally, internal consistency was adequate, as were reliability and error, though notably less than that found in earlier studies.

### Context within the current literature

In this sample, 70% of participants were female which was in contrast to the Chinese sample (25%) [11] and Persian (46%) [10], but closer to the Spanish (58%) [12], Turkish (58%) [14], Polish (60%) [15], and English language (57%) [1] versions, but closest to the Korean (63%) [13]. This Brazilian bias may be explained by the recognized higher prevalence of chronic pain reported by women in Brazil [31], and their higher use of health services in terms of frequency for preventive and diagnostic purposes [32]. Moreover, the mean pain intensity observed in this study (NPRS=5.86) is similar to that reported in previous Brazilian chronic pain [28, 33] and PROM pain validation studies [34]. In terms of the spine region, the low back was the most affected (49%), followed by the thoracic (31%) and cervical (20%) regions. This parallels the findings in the original SFI (50%) [1], Turkish (53%) [14], Chinese (52%) [11], Polish (50.7%) [15], and Spanish (49%) [12] studies. In contrast, in the Persian version, the neck (50%) was the most affected region, followed by the lower back (38.8%) [10].

Regarding the factor loading of the SFI-10-Br, items11 (0.45) and 20 (0.55) were lower than the remaining eight items. Item 11 is related to sleep, and with the lowest factor loading, suggests this was the action least affected by pain. However, most people living with chronic pain do suffer sleep disturbances [35], being 18 times more likely to develop insomnia. There is a recognized bidirectional relationship between sleep and pain [36], where sleep disturbance is an important factor in pain prognosis. This study’s finding could potentially be explained by the relatively low age (29 years) and moderate chronicity (48 months). In contrast, item 20 is associated with sitting. This low factor-loading may be related to the sample diversity, in terms of the spine regions affected by pain, and that sitting is less affected for individuals with neck and thoracic pain (i.e., 51% of the sample). However, it is important to note that prolonged sitting is well recognized at inducing neck pain [37]. A further consideration may be that sleep and sitting, with loadings that substantially exceeded the accepted 0.30 cut off, are simply the least of the 10 actions affected, but more important to the study participants than the 15 items not retained within this shortened PROM.

In the factor analysis of the original 25-item SFI, significant variance in the factor structure was reported and a shortened version recommended [1] (i.e., English [1], Spanish [12], Persian [10], Turkish [14], & Chinese [11]). The Chinese version evaluated the content validity of the SFI to verify the need to remove redundant items, but the response trends and total item correlations showed this was not required [11]. Similarly, the Persian version was subjected to CFA with inconclusive results within the methodological analysis and sample size limitations such that the factorial structure was neither confirmed or denied [10]. In contrast, the Spanish [12], Polish [15], Korean [13], and Turkish [14] SFI versions did not perform CFA.

For the construct validity, the high negative correlation with the RMDQ-g (rho=-0.777) parallels the spine regional PROM findings for the Turkish (ODI, r=0.71; FRI, *r*=0.52) [14], Chinese (FRI, *r*=0.85; ODI, *r*=0.75) [11], Spanish (RMDQ, *r*=0.79) [12], Persian (RMDQ, *r*=0.69) [10], Polish (ODI, *r*=0.82; NDI, *r*=0.82) [15], and English (FRI, *r*=0.85) [1] studies. Similarly, high SFI-10-Br correlation in this study with the SF-36 functional capacity domain (rho = 0.718) was superior to the correlations observed in the Spanish (*r*=0.46) [12], Polish (*r*=0.42) [15] and Chinese (*r*=0.70) [11] studies.

The SFI internal consistency finding in this study was high (α=0.86) which indicates the questionnaire items correlate adequately (i.e. measure the same construct) [38]. Similarly, other studies found adequate α values in the Turkish (α=0.85) [14], Persian (α=0.81) [10], Spanish (α=0.85) [12], Chinese (α=0.91) [11], and English (α=0.91) [1] studies. For reliability, the ability to consistently reproduce a result [18], the finding was high (ICC_2,1_=0.82), indicating coherence, precision, stability, equivalence, and homogeneity [18]. Although notably lower than that of the original SFI-10 and 25-item SFI (ICC_2,1_=0.97), the Chinese (ICC_2,1_=0.96), Turkish (ICC_2,1_=0.95), Persian (ICC_2,1_=0.96), Spanish (ICC_2,1_=0.96), and Polish (ICC_2,1_=0.97) versions, reliability did substantially exceed the required minimum cut off (>0.75) [29]. The SEM and MDC_90_ findings are calculated directly from, and dependent on, reliability. Consequentially, the MDC_90_ (25.15%) is markedly higher than found for the SFI-25 English (MDC_90_=6.4%), Polish (MDC_90_=7.3%), Persian (MDC_90_=4.6%), Turkish (MDC_90_ =7.1%), and Spanish (MDC_90_=6.9%); and that of the SFI-10 English (MDC_90_=9.0%) and extracted Polish (~10.5%). The reasons for this difference in reliability and subsequently the error, is potentially due to: the sample being chronic patients with the time between tests being seven days, as opposed to three days in some acute studies; the sample size (*n*=43) being smaller than that of other studies such as the SFI-10 (*n*=104), the original SFI-25 (*n*=56) and the Polish (*n*=210) but exceeded the Persian (*n*=31); and that recruitment and PROM completion was achieved completely online with no face-to-face or health professional interaction or feedback. However, this does not fully explain the differences as chronic patients and a seven-day retest times were used in both the Spanish and Turkish SFI-25 studies. Perhaps it is due to the sample itself and cultural variations. In either case, this substantial difference will need to be reviewed in further SFI-10 research.

The SFI benefits are not limited to monitoring treatment effectiveness, as multiregional spinal pain is a common problem with significant prevalence in the general population [39]. In light of this, the SFI-10 could be used to gradually replace other single-region spine PROMs with uncertain factor structure, and as a first contact tool to screen, identify, predict, and detect spine-related disorders. Consequently, the SFI-10 as a shortened version will optimize the clinical and scientific applicability, since it reduces the number of errors and the administration time, while maintaining the same quality of information obtained.

Overall, this new shorter 10-item version improves not only the psychometric validity but also the practicality from a reduction in the burden for both the respondent and the health professional. This is consistent with the SFI-10 development and validation study findings [40], and extremely significant, as practicality and clinical burden, the time required to complete and score a PROM, is reported by professionals as one of the most significant barriers to PROM use in routine clinical patient care [41]. These findings should contribute to the efficiency of using a single whole-spine questionnaire to assess patients with pain and dysfunction in one or more spine regions, and to guide future research on this topic.

### Strengths and clinical applicability

This study’s strengths were that the internal structure options of the SFI-Br and the shortened SFI-10-Br were both tested using CFA, with a better model fit only in the 10-item version, including lower AIC and BIC values. The shortened SFI-10 CFA results were consistently more favorable than the original 25-item SFI. Further, all construct validity hypotheses were confirmed by the correlations between the SFI-Br-10 and the criteria instruments, which fulfilled and exceeded the COSMIN guidelines 75% minimum [18]. Additionally, the sample population extends the original SFI evidence which has been limited to clinical and rehabilitation centers and not representative of the general population [10]. The current study fills this gap.

### Limitations and prospects for novel studies

The literature supports PROM administration in a variety of ways, this includes in-person surveys, telephone calls, technology-based online surveys, and self-administered surveys [42, 43]. In contrast, data collection for this study was conducted in an online format and consequently no physical examinations were performed. Further, we recommend that future studies further evaluate the SFI-10 reliability and subsequent MDC_90_ and the responsiveness to verify the questionnaire’s ability to detect clinical improvement after treatment and the minimum clinically important difference found in the validation study. Finally, it is suggested that future studies include psychosocial variables in their eligibility criteria, as their influence on individuals with chronic pain is well established.

## Conclusion

The SFI was successfully translated and culturally adapted for use by Brazilians. The SFI-10-Br demonstrated an unequivocal one-factor structure, high construct and criterion validity with adequate internal consistency, reliability, and error. Further research is required to clarify these properties along with longitudinal studies to determine responsiveness and error.

## Implications for rehabilitation

The SFI Brazilian version was successfully produced with the 10-item version;The 10-item version showed an unequivocal one-factor structure;The 10-item version showed high construct and criterion validity, reliability, internal consistency, and satisfactory error.

### Supplementary Information

**Supplementary Material 1.**

## Data Availability

The data and materials in this paper are available from the corresponding author on request (André Pontes-Silva)
