# Supplementary material for: Translation, cross-cultural adaptation, and validation of the 10-item spine functional index (SFI-10) in the Brazilians with musculoskeletal spine disorders
Source: BMC Musculoskelet Disord. 2024 Apr 5;25:266. doi: 10.1186/s12891-024-07406-0 (PMC10996142; doi:10.1186/s12891-024-07406-0)
Supplement: Supplementary file 1 — Supplementary Material 1. [file 12891_2024_7406_MOESM1_ESM.pdf]

SPINE FUNCTIONAL INDEX (SFI-10) DATA:________________

NOME:________________ LESÃO:______________ ☐ CERVICAL ☐ MEIO DAS COSTAS ☐ LOMBAR

POR FAVOR, COMPLETE: sua coluna pode dificultar a realização de algumas coisas que você normalmente faz. Essa lista contém algumas frases que as pessoas usam para descrever tais problemas. Pense em você nos últimos dias. **Se um item o(a) descreve, marque a caixa "Parcialmente" ou "SIM". Se um item não descreve você, marque a caixa "NÃO".**

POR CAUSA DA MINHA COLUNA:

NÃO	Parcialmente	SIM	
☐	☐	☐	1. Eu evito tarefas pesadas (por ex. limpeza, levantar mais de 5 kg, jardinagem, etc.).
☐	☐	☐	2. Eu tenho dor/problema quase o tempo todo.
☐	☐	☐	3. Eu tenho dificuldade com tarefas domésticas ou familiares.
☐	☐	☐	4. Eu durmo mal.
☐	☐	☐	5. Eu preciso de ajuda com cuidados pessoais (por ex. com banho e higiene pessoal).
☐	☐	☐	6. Minhas atividades diárias (trabalho, contato social) estão prejudicadas.
☐	☐	☐	7. Eu preciso de ajuda ou sou mais lento(a) para me vestir.
☐	☐	☐	8. Eu tenho dificuldade em ficar sentado(a).
☐	☐	☐	9. Eu consigo ficar em pé apenas por pouco tempo.
☐	☐	☐	10. Eu tenho dificuldades para me abaixar (por ex. para pegar um objeto no chão ou colocar as meias).

ESCORE SFI: para pontuar, some os pontos:

☐ **TOTAL (pontos SFI).** **100 – (TOTAL x 10) =** ☐ %
